# Interaction Between Stress and Addiction: Contributions From Latin-American Neuroscience

**DOI:** 10.3389/fpsyg.2018.02639

**Published:** 2018-12-21

**Authors:** Angélica Torres-Berrio, Santiago Cuesta, Silvia Lopez-Guzman, Mauricio O. Nava-Mesa

**Affiliations:** ^1^Fishberg Department of Neuroscience, Friedman Brain Institute, Icahn School of Medicine at Mount Sinai, New York, NY, United States; ^2^Department of Microbiology, University of Texas Southwestern Medical Center, Dallas, TX, United States; ^3^Neuroscience Research Group, Escuela de Medicina y Ciencias de la Salud, Universidad del Rosario, Bogotá, Colombia

**Keywords:** addiction, stress, Latin America, biomarkers, mesocorticolimbic pathway, CRF

## Abstract

Drug addiction is a chronic neuropsychiatric disorder that escalates from an initial exposure to drugs of abuse, such as cocaine, cannabis, or heroin, to compulsive drug-seeking and intake, reduced ability to inhibit craving-induced behaviors, and repeated cycles of abstinence and relapse. It is well-known that chronic changes in the brain’s reward system play an important role in the neurobiology of addiction. Notably, environmental factors such as acute or chronic stress affect this system, and increase the risk for drug consumption and relapse. Indeed, the HPA axis, the autonomic nervous system, and the extended amygdala, among other brain stress systems, interact with the brain’s reward circuit involved in addictive behaviors. There has been a growing interest in studying the molecular, cellular, and behavioral mechanisms of stress and addiction in Latin-America over the last decade. Nonetheless, these contributions may not be as strongly acknowledged by the broad scientific audience as studies coming from developed countries. In this review, we compile for the first time a series of studies conducted by Latin American-based neuroscientists, who have devoted their careers to studying the interaction between stress and addiction, from a neurobiological and clinical perspective. Specific contributions about this interaction include the study of CRF receptors in the lateral septum, investigations on the neural mechanisms of cross-sensitization for psychostimulants and ethanol, the identification of the Wnt/β-catenin pathway as a critical neural substrate for stress and addiction, and the emergence of the cannabinoid system as a promising therapeutic target. We highlight animal and human studies, including for instance, reports coming from Latin American laboratories on single nucleotide polymorphisms in stress-related genes and potential biomarkers of vulnerability to addiction, that aim to bridge the knowledge from basic science to clinical research.

## Introduction

Drug addiction is a chronic neuropsychiatric disorder characterized by the compulsive intake of drugs of abuse and the loss of control over this consumption, in spite of the devastating consequences it carries for the individual (American Psychiatric Association, 2013). According to the World Health Organization, drug addiction affects between 3.4 and 6.6% of the population worldwide and represents a major contributor to the global burden of disease and disability (World Drug Report, 2017). It is estimated that 0.4% of the annual deaths are due to drug abuse, with cannabis, cocaine, opioids, and amphetamine as the most frequently consumed illegal drugs worldwide (World Drug Report, 2017). Furthermore, in Latin America, the geographical region that comprises a part of North America (Mexico), Central America, South America, and the Caribbean, there is a strong association between drug use and having a history of violence, sexual abuse, and other stressful and traumatic events (Montoya et al., 2003; Alegría et al., 2004; Vera et al., 2005; Alvarez et al., 2007; Tucci et al., 2010; Frade et al., 2013; SAMHSA, 2016).

Addiction is cyclical: Individuals who suffer from this disorder transition from periods of abstinence to a return to drug use, complicating therapeutic efforts. The *addiction cycle* — the leading theory in the field — posits that chronic drug users go through three types of phases, one characterized by the preoccupation with consumption, with constant obsessing and craving, followed by another stage dominated by binging and intoxication that leads to a period of withdrawal and negative affect (Koob and Le Moal, 2008). This last stage, also referred to as the “dark side of addiction” and characterized by negative emotional states and stress, eventually transitions back to the preoccupation and craving stage, and the cycle begins again. Major efforts have been made to understand which factors contribute to the development of addiction and drug abuse and also to elucidate the mechanism behind the phases of the addiction cycle. Human imaging and preclinical studies indicate that addiction involves maladaptive changes in the brain’s reward system that result from complex gene–environment interactions (Sweitzer et al., 2012; Walker and Nestler, 2018). Importantly, environmental factors such as early exposure to drugs or chronic stress facilitate the acquisition and maintenance of drug-related behaviors in vulnerable individuals, and are strong predictors of addiction relapse (Saal et al., 2003).

Addiction does not only comprise the positively reinforcing effects of drug consumption. Excessive activation of the neural reward system leads to its dysfunction and to hyperactivation of the brain’s stress response (anti-reward), resulting in an increase in reward thresholds (reward deficit and stress surfeit). Stress therefore plays a very prominent role in all stages of the addiction cycle but particularly in the negative affect phase, also dubbed *the dark side of addiction* (Koob and Le Moal, 2005). However, there is still a gap in the literature regarding the precise molecular and neurophysiological mechanisms that are at play in the interaction between stress and addiction, and how these relate to the behavior exhibited by individuals with this disorder. In order to fill this gap, different laboratories in Latin America have been advancing research that focuses on every level of this relationship, from the molecular pathways of stress and their deleterious effects on the reward system, to the genetic and epigenetic factors that may facilitate these effects. The works of Latin American laboratories seem to not be as strongly acknowledged by the broad scientific audience as studies coming from developed countries (Meneghini et al., 2008). For this reason, here we wish to showcase these Latin American scientific contributions.

This review is the first attempt at compiling a series of studies conducted by Latin American-based neuroscientists, that have devoted their careers to studying the interaction between stress and addiction, from a neurobiological and clinical perspective. For this purpose, we included manuscripts that have been peer-reviewed and published in indexed journals, in which the leading or corresponding authors are affiliated to a university or institution located in Latin America. In the first section, we introduce the neurocircuitry associated with addiction, with a special focus on the mesocorticolimbic system, and we present the most commonly used animal models of drug addiction. In the second section, we briefly describe the role of stress as a main risk factor for drug addiction. In the third section, we report the different contributions made by Latin American researchers who study the molecular and cellular role of stress in drug abuse and addiction using rodent behavioral models. In the fourth section, we emphasize promising preclinical findings coming from Latin America, that could guide novel therapeutic advances for drug addiction. Further, we cover a number of human studies conducted in Latin American population that relate SNPs in stress-related genes to drug addiction vulnerability. Finally, we provide a concluding perspective and discuss opportunities for new research on which Latin American neuroscience could take the lead.

## Drug Addiction: Established Mechanisms and Animal Models

**FIGURE 1:**
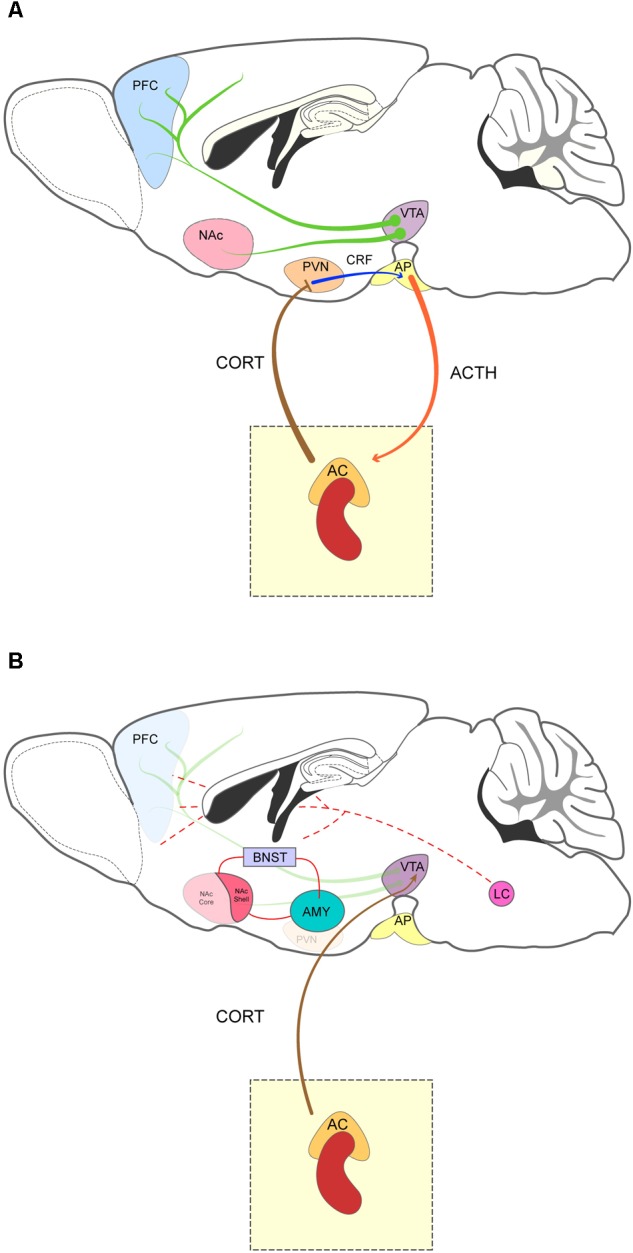
Sagittal representation of the mesocorticolimbic system and the brain stress-related areas. **(A)** The mesocorticolimbic system is composed of dopamine projection neurons (green arrow) that innervate the nucleus accumbens (NAc) and prefrontal cortex (PFC). Drugs of abuse such as cocaine or amphetamine increase the activity of VTA neurons and lead to dopamine release in the NAc and PFC. The HPA axis comprises the paraventricular nucleus of the hypothalamus (PVN), the anterior pituitary gland (AP) and the adrenal cortex (AC). Stressful stimuli activate the PVN, which releases CRF (blue arrow) in the AP and stimulates adrenocorticotropic hormone (ACTH) (orange arrow) secretion into the bloodstream. In turn, ACTH reaches the AC to induce release of corticoids (CORT, brown arrow). Activation of the HPA is terminated by a negative feedback mechanism induced by corticoids in the PVN and AC. Acute and chronic exposure to stress lead to vulnerability to drug addiction by strengthening plasticity across the mesocorticolimbic pathway. Indeed, stress induces DA release in the PFC and NAc and “*sensitizing*” VTA neurons (Saal et al., 2003). **(B)** Relevant stress-related areas and their interaction with the brain’s reward system. Corticosteroids from the adrenal gland stimulate the VTA and the extended amygdala [bed nucleus of the stria terminalis (BNST), amygdala (AMY), and the shell of the NAc]. The arousal system and the noradrenergic projections from the locus coeruleus (LC) is also represented (dashed lines).

The brain’s reward system comprises several regions that are activated in response to rewarding stimuli — such as food, water, or sex — and constitutes an important regulator of complex cognitive processes, like motivation, expectations, and emotions (Koob and Le Moal, 2005; Volkow et al., 2017). This system involves primarily the mesolimbic and the mesocortical DA pathways as well as the medial forebrain bundle, and the extended amygdala (Koob and Le Moal, 2005; Lüscher and Malenka, 2011). The mesolimbic pathway includes DA projections that originate in the VTA and innervate primarily the MSNs of the NAc. The mesocortical pathway, on the other hand, comprises the projections of VTA DA neurons to the PFC (Figure 1A) (Lüscher and Malenka, 2011; Lammel et al., 2014). Release of DA by VTA neurons is further regulated by the PFC and the NAc. Pyramidal neurons in the PFC and NAc MSNs innervate VTA GABAergic interneurons which, in turn, exert an inhibitory control over DA neurons of the VTA (Carr and Sesack, 2000). For a more comprehensive review, see Lüscher and Malenka (2011).

Acute intake of drugs of abuse leads to rapid activation of DA neurons in the VTA and results in DA release in target areas. Imaging studies report that healthy individuals exhibit increased DA efflux in the NAc after acute alcohol and amphetamine consumption (Leyton et al., 2002; Boileau et al., 2003). These findings are consistent with preclinical evidence from rodents exposed to acute administration of cocaine, heroine, or endocannabinoids (Kalivas and Duffy, 1990; Weiss et al., 1992). In addition, repeated exposure to drugs leads to long-lasting maladaptive changes in the mesocorticolimbic system, rendering individuals more vulnerable for the development of addiction and relapse (Saal et al., 2003; Hyman et al., 2006). For example, cocaine users display increased DA release in the PFC and striatum in response to craving-inducing drug associated cues, suggesting that complex context-drug associations activate the mesocorticolimbic system during craving (Volkow et al., 2009). Furthermore, increases in the expression of monoamine transporters have also been observed in chronic cocaine and opioid users (Malison et al., 1998; Kish et al., 2001; Crits-Christoph et al., 2008; Shi et al., 2008).

The use of animal models has become a tremendous tool for studying drug-associated behaviors and deciphering the molecular mechanisms underlying the long-term effects of exposure to drugs. Different paradigms such as behavioral drug sensitization, CPP, and self-administration have been developed to study drug-reward, craving, and relapse in rodents. **Behavioral drug sensitization** was introduced to measure the enhanced behavioral and motivational effects induced by repeated administration of stimulant drugs (Vezina and Leyton, 2009). This paradigm is divided in two phases: development and expression. During the development of sensitization, repeated injections of psychostimulants such as cocaine or amphetamine are given intermittently to rodents over a period of several days, while a separate group of animals are injected with the drug’s vehicle (e.g., saline). Expression of drug sensitization is assessed during a drug challenge administered following a period of drug abstinence, and is associated with an enhanced locomotor activity observed in animals previously exposed to the psychostimulant, in comparison to rodents treated with vehicle (Vezina and Leyton, 2009). CPP is a form of appetitive Pavlovian conditioning used to determine the association strength between drugs of abuse and a specific context. The basic procedure consists of two learning stages and one test: first, the drug of interest (e.g., cocaine or heroin) is administered in a particular environment (e.g., a chamber with white walls). In the second stage, a different environment (e.g., a chamber with black walls) is paired with the absence of the drug (e.g., vehicle administration). During the test, the time spent in each of the environments in the absence of the drug is recorded. Rodents that exhibit heightened place preference spend significantly more time in the drug-paired compartment and are therefore responding to the rewarding effects of the drug (Prus et al., 2009). Finally, **self-administration** is a behavioral procedure used to assess *voluntary* drug-intake in rodents. This paradigm is based on operant learning in which drug-naïve rodents are trained to perform a specific behavior (e.g., lever pressing or nose-poking) that is contingent on the availability of the drug, thus making the drug a reinforcer of that behavior (Panlilio and Goldberg, 2007). Drug self-administration is particularly useful to studying processes such as drug motivation, extinction, and reinstatement (relapse) following an abstinence period (Panlilio and Goldberg, 2007). It is important to mention that certain situations — such as stress or drug exposure — can enhance the subsequent behavioral or neural responsiveness to a different addictive drug (Berridge, 2004). This phenomenon, known as **cross-sensitization**, can be observed in the three behavioral paradigms described above. In subsequent sections, we will discuss the remarkable work of Latin American neuroscientists who make use of these behavioral models to understand the neurobiological mechanisms of addiction.

## Stress as a Risk Factor for Drug Addiction

Stress is a neurophysiological and behavioral response that allows organisms to respond to environmental challenges perceived as threatening (Koolhaas et al., 2011). This process is directly controlled by the HPA axis and the autonomic nervous system, top-down systems that prime an organism for *fight-or-flight* or freeze responses, and contribute to restoring homeostasis (Koolhaas et al., 2011). Upon exposure to a stressor, the PVN of the hypothalamus secretes corticotropin-releasing factor (CRF), into the anterior pituitary gland, where it stimulates the release of ACTH into the bloodstream. As a result, ACTH reaches the adrenal cortex where it binds to its receptors to induce secretion of corticosteroids. Once in the bloodstream, corticosteroids are transported to different target organs where they bind to MR and GR receptors. Corticosteroids also induce a mechanism of negative feedback, suppressing the release of CRF and ACTH and ceasing the stress response (Figure 1A) (Carrasco and Van de Kar, 2003; McEwen, 2007).

Epidemiological studies have identified stress as one of the main risk factors for the development of drug addiction, and as a strong predictor of high craving and relapse to drug use (Sinha, 2008; Mantsch et al., 2016). Both cocaine and opioid-dependent individuals report having experienced stressful life events prior to drug seeking or relapse (Khantzian, 1985; Sinha, 2008). They exhibit increased levels of stress-related hormones, including CRF and ACTH at baseline or after pharmacological and psychological challenges (Schluger et al., 2001; Contoreggi et al., 2003). Preclinical models of addiction have demonstrated that exposure to stress can enhance the rewarding effects of drugs and the acquisition of drug-seeking behaviors. In this perspective, stress facilitates the formation of associations between contextual information and drugs (Mantsch et al., 2016).

In addition to the HPA axis, other relevant brain structures mediate the stress response and are involved in the pathophysiology of drug dependence, including the central and medial nucleus of the amygdala, the BNST, and the medial NAc (together known as the extended amygdala) (Figure 1B) (Alheid and Heimer, 1988; Koob, 2009). These areas are key substrates for the feedforward interaction between CRF and NE. CRF activates NE release in the locus coeruleus (Valentino et al., 1991) which in turn increases CRF in the extended amygdala. These two factors jointly participate in the progression of drug consumption from a positively reinforcing experience to a relief-seeking behavior from the negatively reinforcing effects of withdrawal (Heilig and Koob, 2007; Koob, 2009). NE induces CRF release and this cross-talk between both systems participates in the negative emotional states and drug-seeking behavior associated with drug withdrawal (Koob, 2009). NE is involved in the arousal-promoting effects of certain drugs like psychostimulants, but it has also been implicated in the increase in drug-cue salience and the attentional bias that are known to contribute to relapse (Chaijale et al., 2015; España et al., 2016). The interaction between PVN-CRF and midbrain DA systems in the VTA also contributes to the stress response associated to addictive behavior (Kelly and Fudge, 2018). Recent evidence indicates that projections from the BNST to the VTA requires the signaling of CRF receptor type 1 (CRFR1) and receptor type 2 (CRFR2) to modulate binge-like ethanol drinking (Rinker et al., 2017).

Several laboratories located in Latin America have contributed to the understanding of how acute or chronic stress, associated with exposure to addictive drugs, lead to plastic changes in the brain’s reward system and increase vulnerability to addiction and relapse. In the following section, we describe this very diverse work, which spans the molecular and cellular levels, focusing on different pathways affected by stress mediators, and also explores the behavioral and clinical consequences of stress on addicted populations.

## Contributions From Latin America

### Stress and Psychostimulants: Amphetamine and Cocaine

For more than two decades, the laboratory of Dr. Liliana Cancela, at the *National University of Córdoba*, Argentina, has worked on characterizing the molecular mechanisms behind the increased vulnerability to developing substance use disorders observed in previously stressed subjects. Specifically, using rats as an animal model, Dr. Cancela’s group has focused on the effects of acute and chronic restraint-induced stress on the subsequent response to drugs of abuse, including amphetamine, cocaine, and morphine. We describe here her work on the psychostimulants amphetamine and cocaine.

#### Amphetamine

In a series of studies published between 1997 and 2007, Dr. Cancela’s group evaluated the effects of acute and chronic restraint stress on the rewarding and stimulating properties of amphetamine (Kalivas and Duffy, 1990; Díaz-Otañez et al., 1997; Pacchioni et al., 2002, 2007). Using behavioral drug sensitization, Dr. Cancela’s group revealed that both acute and chronic restraint stress lead to increased locomotor activity (sensitization) after a subsequent challenge with different doses of amphetamine. The authors showed that systemic administration of antagonists against D1, D2, or opioids receptors performed before acute restraint exposure, suppressed the subsequent stress-induced sensitization to amphetamine. These findings reveal an opioid-DA interaction underlying stress-induced behavioral sensitization (Díaz-Otañez et al., 1997). Moreover, in 1999 Cancela’s group reported that exposure to acute, but not chronic stress, led to an increase in the time the animals spent in the drug-paired compartment during a CPP paradigm, a measurement of the rewarding properties of amphetamine. Interestingly, while stress-induced increase in the rewarding properties of amphetamine depends on the activations of the D1 and/or D2 DA receptors, this effect is independent from the opioid system (Capriles and Cancela, 1999) contrasting with their previous finding on stress-induced behavioral sensitization (Díaz-Otañez et al., 1997). Together, these two publications demonstrate that stress alters opioid-DA signaling, modifying the subsequent locomotor and rewarding properties of amphetamine.

Dr. Cancela’s group extended their findings by studying the interaction between DA and glutamate signaling. To this end, the authors chose *in vivo* brain microdialysis, a technique that allows to sample extracellular fluids by means of an implanted hollow fiber constructed from a dialysis membrane (Westerink, 1995), to determine neurotransmitter levels in discrete brain areas. They found that acute stress-induced behavioral sensitization to amphetamine, previously reported (Díaz-Otañez et al., 1997), was related to a DA overflow in all the striatal nuclei. More specifically, there was a significant increase in DA release after an amphetamine challenge in the NAc core, NAc shell, and dorsal striatum, 24 h after a single stress exposure in comparison to non-stressed animals (Pacchioni et al., 2002, 2007). Remarkably, 8 days after restraint-induced stress, the enhanced DA release was maintained only in the NAc core (Pacchioni et al., 2007). Regarding glutamate transmission, they showed that stress-induced behavioral sensitization to amphetamine is prevented by a systemic administration of an NMDA receptor antagonist before stress exposure (Pacchioni et al., 2002, 2007). Moreover, this systemic NMDA blockade also prevented the increase in DA release (Pacchioni et al., 2002, 2007). Altogether, the evidence presented in these reports revealed that stress-induced behavioral sensitization to amphetamine is mediated by a sensitized response to the psychostimulant, at the level of dopaminergic neurotransmission in the different nuclei of the striatum, as well as by NMDA-glutamatergic receptor activation.

#### Cocaine Sensitization

Using the restraint-induced stress model, Dr. Cancela’s laboratory also sought to determine the molecular mechanism mediating the cross-sensitization induced by stress on the locomotor effects of a cocaine injection in adult rats. In a study published in 2012, the group evaluated the effects of repeated stress on actin cytoskeleton remodeling in the NAc and PFC, and assessed their role in the expression of cross-sensitization to cocaine-induced locomotor activity (Esparza et al., 2012). To this end, the authors exposed adult rats to 2 h restraint stress, for seven consecutive days. Three weeks after the completion of the restraint protocol, they challenged these animals with a cocaine injection. The results showed that repeated stress exposure was associated with changes in proteins that regulate the actin cytoskeleton in the NAc and PFC. Remarkably, a microinfusion of Latrunculin A, an inhibitor of actin polymerization, performed before the cocaine challenge into the NAc but not the PFC, was sufficient to inhibit the expression of cocaine cross-sensitization. Similarly, changes in postsynaptic morphology after acute exposure to cocaine were found exclusively in the NAc. AMPA receptor (AMPAR) surface expression in the NAc increased after cocaine exposure in previously stresses animals, a process that was also blocked by latrunculin A. Glutamate inhibition by the antagonist CNQX prevented the stress-induced sensitized response. Taken together, these findings suggest that restraint-induced stress modifies the surface expression of the AMPAR triggered by a cocaine challenge, by altering the actin dynamics in the NAc, giving rise to the behavioral cross-sensitization phenomenon.

In their next paper, published in 2016, Dr. Cancela’s group revisited the effects of acute stress in the response to psychostimulants later in life, using a longer period — 3 weeks — between the acute stress experience and the exposure to the drug (Garcia-Keller et al., 2013). With this work, the authors were able to dissect for the first time the role of the subdivision of the NAc (*core* and *shell*) in the behavioral cross-sensitization to cocaine. By mean of microdialysis probes located in the different subregions of the NAc, Garcia-Keller et al. (2013) demonstrated that acute restraint-induced stress leads to an enhanced behavioral response to a cocaine challenge due to an exacerbated release of DA and glutamate in the *core*, but not in the *shell*, of the NAc. More specifically, the expression of behavioral sensitization to cocaine after stress depended on the activation of the AMPAR in the NAc *core*. Indeed, the stimulation of AMPAR in the *core* of the NAc of pre-stressed animals was sufficient to trigger an enhanced behavioral response in comparison to non-stressed animals. As pointed out by the authors, all these changes induced by stress resembled the enduring plastic modifications observed after repeated exposure to cocaine, and support the idea that stress leads to a facilitation of the processes involved in the development of cocaine addiction in animal models.

Finally, in another study the authors explored the relationship between acute stress and the rewarding properties of cocaine (De Giovanni et al., 2016). More specifically, they explored the reinstatement of an extinguished cocaine-induced CPP by exposing the animals to acute restraint-induced stress. The authors found that glutamate NMDA receptors were involved in the development and the expression of cocaine-CPP reinstatement after restraint-induced stress. Interestingly, microinfusions of the NMDA antagonist MK 801 in the *core* of the NAc but not the *shell* prevented this reinstatement, suggesting the core of the NAc is key for the behavioral expression of the rewarding effects of cocaine.

With all these studies, using a combination of behavioral and biochemical techniques, Cancela’s group has contributed specific evidence of the molecular mechanisms involved in the synergistic effect of stress on psychostimulant drug addiction and relapse. While not explored yet by Dr. Cancela’s laboratory, one interesting possibility is that all these synergistic effects between stress and psychostimulants, are mediated by a neurotransmitter-release-modulation due to stress-induced alterations in CRF levels (Koob, 2015; Kelly and Fudge, 2018).

### Corticotropin-Releasing Factor (CRF) and Cocaine Addiction

Corticotropin-releasing factor, also known as corticotropin-releasing hormone (CRH), is a major neuropeptide that regulates the endocrine and behavioral response to stress (Bale and Vale, 2004). CRF is member of a larger family of peptides that also include CRF binding protein (CRF-BP), UCN I, UCN II, and UCN III, as well as two GPCRs, CRFR1 and CRFR2. Cell bodies that express CRF are located in brain areas involved in the stress response, such as the PVN, the extended central amygdala, the basal forebrain, and the brainstem (Swanson et al., 1983; Kelly and Fudge, 2018). CRFR1 is highly expressed in the cerebral cortex, cerebellum, medial septum, and anterior pituitary (Radulovic et al., 1998), whereas CRFR2 is detected in the LS, ventromedial hypothalamus, and choroid plexus (Chen et al., 2005). During the stress response, CRF binds preferentially to CRFR1 in the anterior pituitary to activate the HPA axis (Bale and Vale, 2004).

Preclinical models have demonstrated an important role for CRF in the susceptibility to drug addiction (Corominas et al., 2010; Koob and Zorrilla, 2010). Indeed, CRF administration increases cocaine-induced locomotor activity (Sarnyai et al., 1992), enhances amphetamine-induced stereotypic behaviors (Cole and Koob, 1989), and promotes relapse to cocaine seeking in rodents (Erb et al., 1998). In contrast, CRFR1 antagonists prevent stress-induced CPP to cocaine (Lu et al., 2001) and sensitization to amphetamine (Cole et al., 1990). These behavioral alterations are associated with changes in the expression of *Crfr1* and *Crf* mRNA in areas such as the NAc or amygdala (Hansson et al., 2007; Sommer et al., 2008). Notably, humans with SNPs in the *CRH-BP* and *CRHR1* genes who have experienced adverse life events exhibit higher risk to alcohol consumption in comparison to non-carriers (Blomeyer et al., 2008; Enoch et al., 2008). In addition, SNPs in the *CRH-BP* gene have been related to cocaine and heroin abuse (Levran et al., 2014), further supporting the role of the CRF family in addiction.

The laboratory of Dr. Katia Gysling at the Department of Cellular and Molecular Biology of the *Pontifical Catholic University of Chile*, has extensively studied the mechanisms by which drugs of abuse induce plastic changes in the rodent brain, as well as the role of stress in promoting vulnerability to addiction-like behaviors. Specifically, her studies have been focused on understanding how CRF modulates the connection between the LS and the VTA in response to drugs of abuse (Sotomayor et al., 2005; Sotomayor-Zárate et al., 2010, 2013; Renard et al., 2014). The LS is a brain nucleus located in the subcortical forebrain. The LS is highly innervated by VTA DA neurons and sends GABAergic projections to the NAc, lateral hypothalamus, and VTA, thus contributing significantly to reward and motivation processes (Renard et al., 2014).

Using pharmacological manipulations and microdialysis, the Gysling laboratory has demonstrated that drugs of abuse such as amphetamine, morphine, or cocaine increase DA extracellular levels in both the VTA and LS (Sotomayor et al., 2005; Sotomayor-Zárate et al., 2010, 2013; Renard et al., 2014). Remarkably, this effect can be modulated either by stress exposure or by direct activation of CRFR1 in the LS (Sotomayor-Zárate et al., 2013, 2015). To illustrate this connection, Sotomayor-Zárate et al. (2013, 2015) from Gysling’s group, reported that activation of CRFR1 increased DA release in the LS of saline-treated rats. However, repeated treatment with cocaine prevented CRFR1-induced DA levels in the LS at short and long-terms after drug withdrawal, indicating that cocaine exposure results in long-lasting alterations of the DA release to the LS (Sotomayor-Zárate et al., 2013). These findings suggested that CRF-CRFR1 signaling initiated by stress impairs LS function and, in turn, leads to altered VTA activity. In this regard, Sotomayor-Zárate et al. (2013), later reported that rats injected with cocaine displayed elevated DA levels in the VTA as compared to saline-treated rats. Interestingly, repeated exposure to stress by immobilization, or local infusion of CRF, blunted cocaine-induced DA levels. Furthermore, blocking CRFR1 increased DA levels in the VTA of rats previously exposed to repeated stress by immobilization (Sotomayor-Zárate et al., 2015). More recently, Vega-Quiroga et al. (2018), showed that stimulation of the LS increased DA levels in the VTA by innervating GABAergic interneurons and decreasing GABA-induced inhibition of DA neurons (Vega-Quiroga et al., 2018). Together, these results demonstrate that stress determines DA levels in the LS and VTA in response to cocaine by regulating CRF binding on CRFR1.

Another important contribution from Dr. Gysling’s group was to provide the first report that CRF-BP and the α isoform of CRFR2 are co-expressed in VTA synaptosomes innervating the LHA. Importantly, these VTA innervations are glutamatergic and GABAergic. Similar to the LS, the LHA regulates motivation as well as feeding and drinking behaviors (Stuber and Wise, 2016). Using a protein–protein interaction model, Dr. Carlos F. Lagos in collaboration with Dr. Gysling, predicted the interaction between CRF-BP and the α and β isoforms of the CRF2R, and revealed that CRF-BP exhibited higher affinity for the CRF2αR isoform (Slater et al., 2018). Indeed, CRF-BP functions as an escort protein that allows the trafficking of CRF2αR from the intracellular space to the cell surface (Slater et al., 2016). Previous evidence demonstrated that reinstatement of cocaine self-administration induced by foot-shock stress was blocked by the infusion of CRF2R antagonists in the VTA (Wang et al., 2007). This effect is mediated by CRF-BP, which leads to DA and glutamate release and potentiates NMDA-dependent synaptic plasticity in the VTA (Ungless et al., 2003; Wang et al., 2007). Taken together, these findings suggest that alterations of the CRF-BP-CRF2R signaling in the VTA-LHA pathway can also contribute to the enhanced effects of stress on cocaine addiction and stress-induced relapse, and introduces the CRF2αR isoform as a potential therapeutic target. Future studies involving pharmacological manipulations in the VTA and microdialysis in the LHA in rodents exposed to psychostimulants would be required to causally evaluate this hypothesis.

### Molecular Studies: The Role of Wnt/β-Catenin Pathway

Wnt factors are a family of secreted signaling molecules that have been associated with different processes ranging from cell fate determination during embryonic development, cellular polarity, cell proliferation, cell cycle arrest, differentiation, apoptosis, and tissue homeostasis (Oliva et al., 2013, 2018). During development of the central nervous system the Wnt signaling pathway is implicated in a wide spectrum of physiological processes, including neuronal connectivity and synapse formation. Wnt proteins and components of the Wnt pathway are expressed in the brain since early development to adult life, however, little is known about its role in mature synapses. Here, we review evidence indicating that Wnt proteins participate in the remodeling of pre- and post-synaptic regions, thus modulating synaptic function. We include the most recent data in the literature showing that Wnts are constantly released in the brain to maintain the basal neural activity. Also, we review the evidences that involve components of the Wnt pathway in the development of neurological and mental disorders, including a special emphasis on *in vivo* studies that relate behavioral abnormalities to deficiencies in Wnt signaling. Finally, we include the evidence that supports a neuroprotective role of Wnt proteins in Alzheimer’s disease. We postulate that deregulation in Wnt signaling might have a fundamental role in the origin of certain neurological diseases, by altering the synaptic function at stages where the phenotype is not yet established but when the cognitive decline starts (Oliva et al., 2013). Dysregulation of the Wnt pathways is related to multiples diseases including degenerative disorders, cancer, and psychiatric conditions (Ciani and Salinas, 2005; Oliva et al., 2018). Once secreted to the extracellular space, Wnt factors interact with specific receptors to activate one of three ‘cascades’: (1) the canonical Wnt or Wnt/β-catenin pathway, which acts through the transcriptional activity of β-catenin, plus two β-catenin-independent pathways: (2) the non-canonical planar cell polarity pathway, and (3) the non-canonical Wnt/calcium pathway, each one with specific physiological functions (Oliva et al., 2018).

The laboratory of Dr. Alejandra Pacchioni, located in the *National University of Rosario*, Argentina, has studied the relationship between the canonical Wnt pathway and the effects of cocaine exposure in rats. Dr. Pacchioni’s group revealed that the canonical Wnt pathway is crucial for the cocaine-induced neuroadaptations in the PFC and the NAc that underlie the development and expression of behavioral sensitization (Cuesta et al., 2017a,b). Furthermore, the authors revealed that pharmacological modulation of the canonical Wnt pathway impacts and modifies the long-lasting effects on cocaine-induced neuroplasticity (Cuesta et al., 2017a).

Environmental factors, such as stimulant drugs, disrupt the patterns of neural networks that are still maturing during adolescence by altering crucial developmental signaling proteins (Cuesta et al., 2018). In this regard, more recently, Dr. Pacchioni’s group became interested in evaluating how stress-induced by social isolation early in life confer a vulnerability to develop addiction-like behaviors during adulthood. Using behavioral sensitization and CPP, Cuesta et al. (2018) first revealed that rats exposed to only 5 days of social isolation during adolescence (from PND 30 to 35) increased cocaine-induced CPP and drug sensitization when tested during adulthood, indicating that social isolation early in life enhances the rewarding and stimulating properties of cocaine. Importantly, the authors also examined whether stress-induced by social isolation in adolescence altered the activity of the canonical Wnt pathway, before and after cocaine exposure. They found that social isolation in adolescence is sufficient to modify the levels of β-catenin and glycogen synthase kinase 3β (GSK3β), two main effectors of the Wnt/β-catenin pathway, in the PFC. This change was observed even 10 days after the completion of the social isolation protocol. During adulthood, animals previously exposed to stress in adolescence exhibited a higher behavioral response during the expression of cocaine sensitization, an effect associated to elevated activity of the Wnt/β-catenin pathway in the NAc. Taken together, the studies of Dr. Alejandra Pacchioni’s laboratory describe a novel function of the canonical Wnt pathway as a mediator of the effects of drugs of abuse like cocaine. Her research posits the Wnt/β-catenin pathway as one of the molecular pathways affected by stress that would be involved in increasing later vulnerability to addiction and relapse (Cuesta and Pacchioni, 2017).

### Alcohol Intake, Sensitization, and Stress

There is a strong association between stress and alcohol consumption. Alcohol use is elevated in individuals with high levels of anxiety (i.e., social phobia), whereas some people consume alcohol to reduce anxiety and stress (Kushner et al., 1990; Terra et al., 2006). In this context, stress has a main role in increasing the vulnerability for alcohol abuse and in triggering relapse. A considerable number of research groups in South America has studied the association between stress and alcohol intake using a preclinical approach. For example, Dr. Roseli Boerngen-Lacerda at *Universidade Federal do Paraná* (Brazil) demonstrated that ethanol intake induced an anxiolytic-like effect in mice with high-anxiety (HA) but not in medium-anxiety (MA) mice. However, HA and MA mice displayed a similar preference for ethanol intake when subjected to three different concentrations of ethanol, suggesting that anxiety-related traits were not a determining factor for the acquisition of ethanol intake (Correia et al., 2009). Another study by the same lab revealed that CRF1 receptor signaling in the amygdala is involved in ethanol consumption in “heavy drinking” mice, pointing to a relationship between this stress-mediated mechanisms and alcohol use severity (Correia et al., 2015). Ferraz and Boerngen-Lacerda (2008) reported that increased serotonergic neurotransmission reduced ethanol sensitization induced by chronic-handling stress. They suggested that the protective effects of mianserin (an tetracyclic antidepressant) against stress is possibly mediated by 5-HT2 receptors (Ferraz and Boerngen-Lacerda, 2008).

The group of Dr. Rosana Camarini at Departamento de Farmacologia, *Universidade de São Paulo* (Brazil), has contributed significantly to the understanding of the role of stress in ethanol-induced sensitization. In a recent study, her team showed that cross-sensitization between chronic unpredictable stress and ethanol is mediated by the nitric oxide system (Santos-Rocha et al., 2018). Additional contributions from the Camarini lab and collaborators, include the role of Homer2 (an scaffolding protein system associated with glutamate receptors) in stress-alcohol locomotor cross-sensitization (Quadir et al., 2016), and the main role of housing conditions and other environmental changes that affect stress response and ethanol-induced reward (Araujo et al., 2005; Faria et al., 2008; Rueda et al., 2012; Novaes et al., 2017; Pautassi et al., 2017). Finally, we highlight the studies of Dr. Roberto Frussa-Filho from *Universidade Federal de São Paulo* about environmental modifications (Araujo et al., 2005; Fukushiro et al., 2010) and sleep deprivation (Araujo et al., 2006) in alcohol-induced behavioral effects.

#### Early-Life Stress and Ethanol Consumption: Sex Differences

Dr. Ricardo Pautassi, from the *Instituto de Investigación Médica Mercedes y Martín Ferreyra* (Argentina), has extensively studied how exposure to stress early in life affects ethanol intake. To this end, Pautassi’s team has used a stress model of early maternal separation in rats, from PND 1 to PND 14, and evaluated ethanol consumption immediately after treatment (PND 15) or later in life (adult) (Pautassi et al., 2012; Fernández et al., 2014). His groups reported that early maternal separation led to altered ethanol-induced motivational learning, without affecting ethanol metabolism or ethanol intake (Pautassi et al., 2012). In addition, rats exposed to maternal separation exhibited significantly greater reactivity to the motor stimulating effects of 1.25 g/kg ethanol in comparison to control animals, yet greater motor suppression after 2.5 g/kg ethanol (Fernández et al., 2014). These results revealed that the effects of early life stress on ethanol responses emerged quickly during development. Intriguingly, none of these effects seems to be associated to the kappa opioid receptor system (Pautassi et al., 2012; Fernández et al., 2014), that is thought to mediate some of the aversive consequences of stress (Torres-Berrio and Nava-Mesa, 2018).

In a subsequent set of papers, Dr. Pautassi’s group compared the effects of acute or repeated restraint-induced stress during adolescence and adulthood on ethanol consumption in male and female rats (Acevedo et al., 2013, 2016; Fernández et al., 2016; Wille-Bille et al., 2017). The authors found no effects of acute restraint stress on spontaneous or ethanol-induced locomotion at either age, whereas adult rats showed an enhanced and long-lasting sedative effect due to ethanol exposure as compared to adolescent rats (Acevedo et al., 2013). Intriguingly, 5 days of restraint-induced stress exacerbated free choice ethanol drinking in adolescent, but not adult rats. Furthermore, adult rats had a greater level of ethanol sedation in comparison adolescents rats, but only the adolescent animals exhibited ethanol-induced motor stimulation (Acevedo et al., 2013; Fernández et al., 2016), suggesting that stress modulates the effects of ethanol in an age-dependent manner. Interestingly, Pautassi’s group further reported that female rats that show high baseline anxiety levels displayed higher ethanol intake as compared to female rats with average levels of anxiety. In addition, average-anxiety animals exhibited a significant increase in ethanol intake across sessions after three sessions of restraint stress. Intriguingly, they also found that high-anxiety female rats exposed to stress not only consumed less ethanol than their unstressed counterparts, but also failed to exhibit a significant increase in ethanol consumption across intake sessions (Acevedo et al., 2016), suggesting that individual differences at baseline anxiety levels might account for the effects of stress on ethanol intake in female rats. Finally, in more recent work, the authors exposed adolescent and adult (PND 70) female rats to restrain-induced stress and evaluated ethanol intake. Using similar paradigms for stress and ethanol exposure than in previous papers, they found that restraint stress significantly increased alcohol intake and preference in female adolescent rats. This effect was prevented by administering a single systemic dose of the long-lasting kappa opioid receptor antagonist, norbinaltrophimine, before the first session of restraint. In contrast, stress exposure led to a decrease in alcohol intake and preference in female adult rats (Wille-Bille et al., 2017). Taken together, Dr. Pautassi’s work represents a very innovative contribution to the field, that focuses on how sex differences, developmental stages, and individual factors interact with the effects of stress on alcohol use.

### Cannabinoid System, Stress, and Addiction

Cannabinoids are part of a neuromodulatory system comprising the endocannabinoids anandamide and 2-AG, and the CB1 and CB2 receptors. CB1 receptors are enriched in the brain, including areas such as the hippocampus, basal ganglia, and cerebellum, whereas CB2 receptors are preferentially expressed in cells of the immune system (Mechoulam and Parker, 2013), and sparse localization in VTA dopaminergic neurons and perivascular microglial cells (Núñez et al., 2004; Liu et al., 2017; Zhang et al., 2017). The role of the endocannabinoid system in addictive behavior has been thoroughly reported in preclinical studies (Bilbao, 2013; Gamaleddin et al., 2015; Chen et al., 2017). For example, CB1 receptors modulate reward-related behaviors by affecting VTA and NAc functions (Yazdi et al., 2015), and their expression is upregulated by repeated cocaine administration in the PFC (Blanco et al., 2014). Accordingly, GWAS studies have indicated that the CNR2 gene, which encodes the CB2 receptor, is associated with substance abuse disorders (Liu et al., 2017, p. 2). Moreover, the cannabinoid system modulates the effects of stress on drug-related behaviors such as cocaine acquisition (Miller et al., 2008), alcohol seeking (Onaivi et al., 2008) stress-induced reinstatement to cocaine (Vaughn et al., 2012) and drug relapse (De Vries et al., 2001).

Different laboratories in Brazil have focused on the functional interaction between the glucocorticoid and endocannabinoid systems during learning paradigms, including fear conditioning and drug-induced CPP (Bitencourt et al., 2014; De Carvalho et al., 2014). For example, the team of Dr. Reinaldo Takahashi at the *Universidade Federal de Santa Catarina*, demonstrated that an injection of the CB1 receptor antagonist Rimonabant, impaired reconsolidation of morphine-CPP while the inhibition of anandamide metabolism with URB597 enhanced CPP expression (De Carvalho et al., 2014). In a similar line, studies conducted by the group of Dr. Francisco Guimarães at the Department of Pharmacology of the *University of São Paulo*, have focused on the multifunctional phytocannabinoid, CBD. This molecule weakly antagonizes the CB1 receptor, while serving as an inverse agonist of the CB2 receptor and an inhibitor of Anandamide uptake. In this context, Fogaça et al. (2018), from the Guimarães’ group, reported that CBD induced anxiolytic responses in stressed animals (chronic unpredictable stress), an effect that was blocked by CB1 and CB2 antagonists. Similarly, in a recent study from de Carvalho and Takahashi (2017) rats that acquired morphine or cocaine CPP were reactivated with a single re-exposure to the reinforced compartment. A subset of these animals were treated shortly after reactivation with an injection of CBD and exhibited decreased expression of CPP 1, 7, and 14 days after, suggesting CBD can impair CPP reconsolidation. In another experiment, the authors reactivated CPP and injected CBD in a subset of rats as described before, but then proceeded to complete an 8-day 16 session extinction protocol. After extinction, rats were reinstated with a single dose of morphine and with restraint-induced stress, but only the CBD-treated group showed reduced CPP. Similarly, CBD after reactivation reduced CPP after naltrexone-induced withdrawal. These results indicate that CBD can reduce morphine and cocaine CPP, and this effect seems to be persistent even after a stress-induced reinstatement test (de Carvalho and Takahashi, 2017). These and other studies by Dr. Takahashi and Dr. Guimarães have pointed to the cannabinoid system as an interesting target for the treatment of anxiety-related disorders, substance abuse disorders, and stress-induced relapse (Lee et al., 2017; Stern et al., 2018). In addition, these studies provided neurobiological plausibility in a recent prospective study in a Canadian sample of illicit drug users. In this longitudinal cohort study, intentional use of cannabis was associated with reduced frequency of crack use (Socías et al., 2017). However, Dr. Jimena Frontera and Dr. Alicia Brusco, at the *Universidad de Buenos Aires* in Argentina, have studied the role of the endocannabinoid system in facilitating the reinforcing effects of different drugs of abuse. In an interesting experiment, repetitive exposure to the cannabinoid agonist WIN 55,212-2 (WIN) during early adolescence in mice led to an increase in anxiety-like behaviors, as well as to an enhanced preference for ethanol intake in adulthood (Frontera et al., 2018). This result suggests that the activation of CB1/CB2 receptors could induce anxiety and stress-induced drug seeking behavior in specific periods of vulnerability. The aforementioned studies indicate that endocannabinoid system is an interesting common pathway between emotional states, stress, and addiction.

## Latin American Non-Clinical Studies and Translational Efforts

Latin American researchers have also made significant strides in translating the findings of animal studies on the neurobiological link between stress and addiction to human behavioral and clinical studies. We will end this review by highlighting these efforts and discussing how they may guide future research.

### Studies on Stress and Genetic Vulnerability to Addiction

Understanding how genetic makeup may influence the potential vulnerability or protection for addiction is a topic of great interest to psychiatry. A more specific question is how genes related to the stress response play a role in determining the risk for addiction, and how these genotypes interact with stressful environments. Many regions of Latin America suffer from under-development, scarcity, and high levels of violence, which could represent significant environmental stressors. The laboratory of Dr. Claiton H. D. Bau, at the *Universidade Federal do Rio Grande do Sul* in Porto Alegre, Brazil, has been a steady source of studies on the interaction between stress and different relevant genes to the risk of addiction. Rovaris et al. (2015), from Bau’s team, set out to explore the connection between early life stress, genetic variants of MR and GR receptors, and risk of crack/cocaine addiction, in a genetic association study (Rovaris et al., 2015). They reported an interesting gene–environment interaction between high levels of childhood physical neglect and the rs5522-Val allele of the MR gene (*NR3C2*), which very significantly modulated cocaine addiction severity. Furthermore, the study showed that the gene–gene interaction between *NR3C2* and the GR gene (*NR3C1*) significantly modulated the severity of withdrawal symptoms over time. This finding is in concordance with the reported effect of this and other interactions of the *NR3C1* gene on nicotine addiction severity found in several of their studies (Elkind-Hirsch et al., 1991; Rovaris et al., 2013). These results suggest that stress-response related genotypes, together with a concomitant history of early life stress, determine addiction vulnerability.

In a separate study, also in women addicted to crack cocaine, Bau’s group found that C allele carriers for the *NR3C1* gene rs41423247 SNP had significantly higher depressive symptoms during abstinence (Rovaris et al., 2016). Given the relationship between negative affect and return to drug use, this indicates that this GR genotype could potentially increase the risk for relapse (Heilig et al., 2010; Carelli and West, 2014; Koob et al., 2014). Furthering the case for stress and gene interactions in worsening outcomes and severity of addiction, the group of Dr. Bau reported that variable number tandem repeats of the monoamine oxidase A (MAO-A) promoter — a gene region heavily regulated by glucocorticoids — were related to earlier onset, concomitant drug abuse, and antisocial symptomatology in alcohol use disorder (Contini et al., 2006).

The epidemiological relationship between polysubstance use, defined as the consumption of more than one drug within a specified period of time, and several psychosocial factors (i.e., alcohol use, anxiety symptoms, and family functioning) in a Latin American sample was recently described by the group of Dr. Diego Forero at the *Universidad Antonio Nariño*, in Bogotá, Colombia (Pereira-Morales et al., 2017). In the same laboratory, methylenetetrahydrofolate reductase (MTHFR) methylation levels were found to be associated with perceived stress scores (Cohen’s Perceived Stress Scale), supporting vast evidence of epigenetic changes in stress and psychiatric disorders (Jiménez et al., 2018). In fact, the *MTHFR* C677T polymorphism has been associated with depression in the context of traumatic stress in early life (Lok et al., 2013), again pointing to genetic and epigenetic determinants of stress-induced negative affect. Another genetic study on Colombian population indicated an association between a polymorphism of the *SLC6A3* gene, which encodes the DA transporter or DAT, and addiction to heroin or cocaine (Isaza et al., 2013). Similarly, the same polymorphism was found to be correlated with cocaine dependence in a Brazilian sample (*n* = 699) (Guindalini et al., 2006). These results are in line with the DA dysfunction hypothesis of the pathophysiology of addictive behaviors through the reward system. Interestingly, several types of chronic psychosocial stressors reduce the number of binding sites of DAT in the basal ganglia in animal models (Isovich et al., 2000).

### Biomarkers of Stress-Induced Vulnerability to Addiction

Non-invasive biomarkers are biological characteristics that provide information about normal or pathological processes, and about traits or states associated with a disease (Strimbu and Tavel, 2010). They are crucial diagnostic tools for the identification of individuals who are at higher risk of developing a disorder (*predictors*) or who will greatly benefit from early therapeutic interventions (*mediators*). Biomarkers can range from blood pressure or pulse measurements to complex biochemical analyses of blood and other peripheral fluids, and should be measured in a reliable and reproducible manner (Strimbu and Tavel, 2010). However, one of the current challenges in neuropsychiatry is to find reliable biomarkers of vulnerability to addiction (Kwako et al., 2018).

The laboratory of Dr. Rodrigo Grassi-Oliveira, at the *Pontifical Catholic University of Rio Grande do Sul*, Brazil, has extensively studied the role of early life stress in cocaine addiction and how peripheral levels of stress hormones, transcription factors, and cytokines can serve as potential biomarkers of addiction (Coelho et al., 2014). For example, Grassi-Oliveira et al. (2012) measured the levels of cortisol in hair of women admitted to hospitalization seeking detoxification from crack and cocaine. The authors also recorded the number of stressful events 3 and 6 months prior to hospitalization. According to their results, women with high drug dependence exhibited a positive correlation between hair cortisol concentration and the number of stressful events 3 months before treatment admission, suggesting that hair cortisol provides information about stress response in drug abusers (Grassi-Oliveira et al., 2012). More recently, Levandowski et al. (2016) from Grassi-Oliveira’s group, reported that variations in the levels of cytokines could correlate with addiction treatment and early life trauma. Specifically, the authors reported that the levels of the cytokine TNF-α were significantly lower in cocaine-dependent women at baseline as compared to reference values obtained from healthy women. Interestingly, addicted women who suffered from childhood trauma exhibited higher levels of TNF-α in comparison to those who did not. In addition, while the levels of IL-4 and IL-6 levels approached the reference values after 3 weeks of detoxification in women without trauma, those levels were still elevated in women with a history childhood trauma. Most interestingly, the imbalance between cytokines correlated with severity of withdrawal symptoms 3 weeks after treatment, demonstrating the role of cytokines as indicators of clinical states (Levandowski et al., 2016). Finally, Viola et al. (2014), demonstrated that peripheral levels of growth factors such as GDNF or BDNF were significantly elevated in crack cocaine users during early abstinence. Interestingly, GDNF levels remained elevated after 3 weeks of detoxification in women with a history of childhood sexual abuse (Viola et al., 2014).

### CRF Family as a Potential Pharmacological Target

Animal and human studies on the neurobiology of stress and the pathophysiology of addictive behaviors make CRF an interesting therapeutic candidate (Koob, 2016). Furthermore, the association between SNPs in genes of the CRF family and addiction uncovers its potential role as a predictor biomarker of vulnerability (Enoch et al., 2008; Rovaris et al., 2017). To illustrate this, Rovaris et al. (2017), from the group of Dr. Bau, evaluated whether three different SNPs in the *CRFR1* gene (rs12944712, rs110402, and rs878886) would predict plasma levels of BDNF in addicted individuals. As previously shown, alterations in the levels of circulating BDNF are observed in addiction (Viola et al., 2014, 2015). According to their results, SNPs in the *CRFR1* gene determined circulating levels of BDNF in control individuals, however, this relationship was lost in individuals suffering from cocaine or crack addiction (Rovaris et al., 2017). These results suggest that genetic variants in families of the CRF family and vulnerability to addiction could be readily detected in peripheral fluids.

Clinical trials conducted by the National Institute on Alcohol Abuse and Alcoholism at NIH, Bethesda, MD, United States, have shown that the use of CRFR1 antagonists does not provide therapeutic effects in individuals suffering from alcohol dependence (Shaham and de Wit, 2016; Spierling and Zorrilla, 2017). For example, Kwako et al. (2015) evaluated the ability of pexacerfont, a CRFR1 antagonist, to reduce stress-induced alcohol craving in alcohol-dependent patients during early abstinence. They further assessed HPA activation following a challenge with the synthetic glucocorticoid DEX and with CRF (dex-CRF challenge). The results show that Pexacerfont failed to reduce alcohol craving and suppress HPA axis activation after the dex-CRF challenge in alcohol-dependent individuals (Kwako et al., 2015). In a similar line, Schwandt et al. (2016) administered the CRFR1 antagonist, verucerfont, to anxious, alcohol-dependent women. Again, verucerfont failed to reduce alcohol craving but induced changes in the HPA activation following the dex-CRF challenge (Schwandt et al., 2016). Collectively, these results highlight the urgent need to develop other antagonists of the CRF family to test their potential efficacy in treating alcoholism and drug dependencies, including cocaine or amphetamine. To this point, the research of Dr. Gysling and Dr. Lagos has presented a novel and interesting interaction between the CRF2αR isoform and the CRF-BP, opening the possibility for developing pharmaceuticals that target CRF2αR (Slater et al., 2016, 2018). Their results could guide future human studies that evaluate the potential association between SNPs in CRF2αR and vulnerability to addiction.

### Studies on Addiction, Stress, and Memory Processes

Chronic exposure to drugs of abuse leads to structural changes in key brain areas that support cognitive processes, executive function, and memory. Cocaine users for example, exhibit an accelerated decrease in global gray matter compared to normal aging individuals. This gray matter decline seems to be especially exacerbated in prefrontal and temporal cortices, an observation that suggests chronic cocaine users undergo a “fast-track” brain aging process (Ersche et al., 2013). Dr. Rodrigo Grassi-Oliveira’s laboratory at the *Universidad Católica do Rio Grande do Sul*, tested this proposed phenomenon behaviorally, in a sample of female crack cocaine users compared to healthy aged-matched controls and to healthy older controls (over 60 years of age) who were asked to perform an N-back working memory task. Consistent with the “fast-track” theory, crack cocaine users performed worse than same age controls but no different than older non-users (Sanvicente-Vieira et al., 2016). Moreover, the same laboratory reported that memory deficits in drug users are in fact worsened by early life stress. The authors specifically interrogated a group of crack-cocaine inpatients for any history of childhood maltreatment, that is, any failure of their caregivers to meet their physical, emotional, educational, or healthcare needs during childhood. They found that patients with a positive history of childhood neglect had a significantly lower recall performance in an episodic memory task (Tractenberg et al., 2015). Grassi-Oliveira’s work points to the long-term effects of early life stress in disrupting memory function in individuals with addiction. Interestingly, while these patients may suffer from poor working and episodic memory, drug-related contextual associations seem to be enhanced (Nestler, 2013; Kutlu and Gould, 2016). Indeed, environmental cues are thought to be strong drivers of the reinstatement of learned associations between drug consumption and the context in which it takes place. Stress also plays an important role in these associations. On the one hand, exposure to stress facilitates encoding and retention of rigid and inflexible forms of memory that require associations with an immediate stimulus (Torres-Berrio and Nava-Mesa, 2018), and on the other, stressful environments can reinstate these drug-related memories, facilitating relapse after periods of abstinence. Extinction has been proposed as an intervention that can disrupt this process. It is an active process by which a new inhibitory association is learned, which masks the originally learned association but does not erase it (Bouton, 2004). Dr. Javier Nieto, at the *Universidad Autonoma de Mexico*, has made several contributions to the understanding of how extinction can be enhanced for therapeutic purposes through experiments performed in rodents. His studies suggest that adding a sensory cue at the time of extinction (“cue-exposure”) could reduce spontaneous recovery and reinstatement of the association (Bernal-Gamboa et al., 2017), and that increasing the interval of time in between extinction sessions could also boost its efficacy (Bernal-Gamboa et al., 2018). Clinicians are starting to revisit these findings to incorporate them into relapse-prevention therapies (Havermans and Jansen, 2003).

### Therapeutic Potential of Exercise for Addiction

Animal studies have shown that exercise may reduce stress and addictive symptoms in several models. For instance, forced exercise attenuates nicotine withdrawal syndrome-induced anxiety, depression, and cognition impairment in rats (Motaghinejad et al., 2016). The same effect was also reported in a model of amphetamine relapse (Segat et al., 2014). Similarly, modifications in animals’ housing conditions that facilitate voluntary exercise (e.g., running wheels, ladders, tubes, etc.) seem to have beneficial effects that reduce stress levels (Novaes et al., 2017). These and other EE interventions, such as enhancements in the animals’ sensory, motor, and social stimulation have been studied in the context of substance dependence. A study from Dr. Rosana Camarini at Departamento de Farmacologia, Universidade de São Paulo, (Brazil), showed that EE reduced consumption after acute restraint stress, in rats that had been previously sensitized to ethanol (Marianno et al., 2017). However, the effect of EE on ethanol consumption has shown divergent results. For instance, the same group reported that EE enhanced ethanol’s rewarding effects via an oxytocinergic-dependent mechanism (Rae et al., 2018). The authors suggested this could be explained because chronic oxytocin release induced by EE may have anxiolytic effects that could reduce the aversive effect of initial alcohol intake.

Cabral et al. (2017) at the *Federal University of Rio Grande do Norte* Brazil, found that individuals with substance use disorder had lower baseline metabolic activity in the PFC. Remarkably, one session of aerobic exercise was sufficient to improve PFC oxygenation and inhibitory cognitive control as compared to controls (Grandjean da Costa et al., 2017). They also reported a case of an alcohol use disorder patient in treatment at a psychiatric hospital, who after undergoing a continuous 3-month running program, improved not only in physical performance, but exhibited clear cognitive and autonomic benefits as well (Cabral et al., 2017). Fontes’ group’s work suggests that the addition of exercise to other therapeutic interventions could be effective in minimizing the biological influence of stress on addictive behaviors.

## Conclusion

The vast majority of studies on addiction have been conducted by laboratories located in North America (United States and Canada) and Europe. Indeed, the work of world-leading researchers has inspired a new generation of neuroscientists interested in understanding drug-induced brain alterations. Remarkably, some of the advances in the field of addiction have also been developed by researchers located in Latin America. They have carried forward work on the topic of stress and addiction at every level, from the genetic and epigenetic, to the molecular and cellular, to the behavioral and clinical. Their studies have contributed to our understanding of the influence of stress on addiction vulnerability, of its role in the pathophysiology of relapse, and of opportunities for pharmacological, behavioral, and lifestyle interventions that can ameliorate outcomes for addiction, by counteracting the deleterious effects of stress. Their findings present new potential avenues for research for which Latin America could be at the forefront.

**FIGURE 2:**
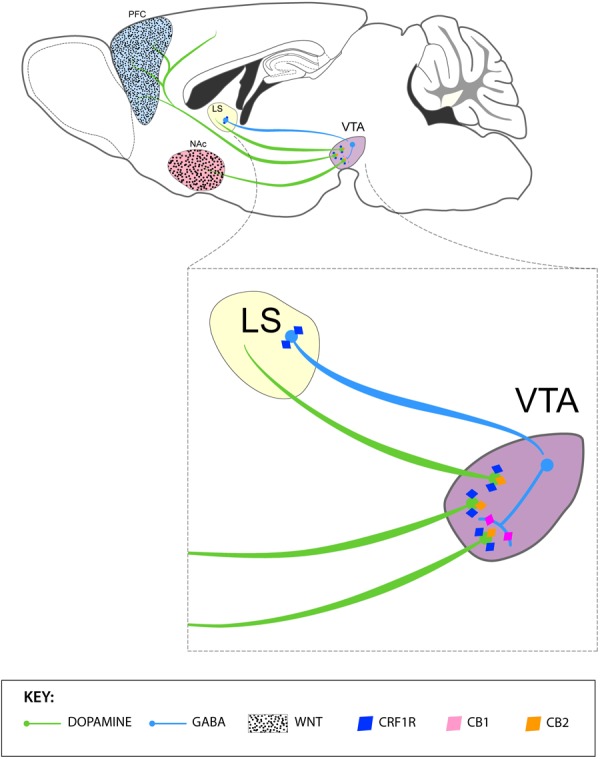
Summary of the contributions by Latin American-based neuroscientist in the neurobiology of stress and addiction. The upper panel depicts VTA DA projections to the PFC and NAc and the modulatory role of GABAergic neurons of the lateral septum (LS) and VTA in the function of DA neurons. Activation of GABAergic neurons of the LS inactivates VTA GABA interneurons and, in turn, disinhibits DA neurons in the VTA. The PFC and NAc express WNT proteins, which play a crucial in the development and expression of cocaine sensitization in rats (black dots). The lower panel represents the expression of *(i)* CRF1 receptors by GABAergic neurons of the LS and DA neurons of the VTA, *(ii)* CB1 receptors GABAergic interneurons of the VTA and *(iii)* CB2 receptors in DA neurons of the VTA.

Based on results largely coming from Latin American laboratories, we highlight the association between SNPs in stress-related genes and addiction. Interestingly, the presence of those SNPs may interact with blood levels of stress hormones, transcription factors, and cytokines, suggesting a promising pathway toward identifying reliable biomarkers of vulnerability to drug abuse, and relapse in Latin American population. Preclinical evidence also points at CRF receptors in LS and the VTA, specifically the CRF2αR isoform, as a potential therapeutic target for drug addiction. We also emphasize their contributions in the study of neuronal plastic mechanisms of cross-sensitization of psychostimulants and ethanol, as well as the identification of the Wnt/β-catenin pathway as a critical neural substrate for the interaction between stress and addictive behavior. Finally, Latin American studies indicate that the cannabinoid system is a promising target for the treatment of anxiety-related disorders and stress-induced relapse. A summary of these non-clinical contributions is represented in the Figure 2.

Taking into account the high prevalence of stress in Latin American population, as well as their higher rates of addiction and comorbidity with stress-related disorders, the study of the mechanisms behind vulnerability and resilience in this specific population can be a very interesting model, that may have impact abroad. Likewise, the development of new specific drugs that target the molecular pathways and receptors described in this review, is very promising, especially for addressing therapeutically the perverse relationship between stress and substance use disorders.

## Author Contributions

AT-B and MN-M designed and supervised the project. AT-B, SC, SL-G, and MN-M wrote the manuscript. All authors discussed the literature and commented and edited the manuscript.

## Conflict of Interest Statement

The authors declare that the research was conducted in the absence of any commercial or financial relationships that could be construed as a potential conflict of interest.

## Abbreviations

2-AG: 2-arachidonoylglycerol
ACTH: adrenocorticotropic hormone
AMPA: α-amino-3-hydroxy-5-methyl-4-isoxazolepropionic acid
BDNF: brain derived neurotrophic factor
BNST: bed nucleus of the stria terminalis
CB: cannabinoid receptors
CB1: cannabinoid type 1 receptor
CB2: cannabinoid type 2 receptor
CBD: cannabidiol
CNQX: 6-cyano-7-nitroquinoxaline-2,3-dione
CPP: conditioned place preference
CRF: corticotropin releasing factor
CRF-BP: corticotropin releasing factor binding protein
CRFR1: corticotropin releasing factor receptor 1
CRFR2: corticotropin releasing factor receptor 2
CRH: corticotropin releasing hormone
*CRHR1*: corticotropin releasing hormone genes
DA: dopamine
DEX: dexamethasone
EE: environmental enrichment
GDNF: glial cell derived neurotrophic factor
GPCRs: G protein-coupled receptors
GRs: glucocorticoid receptors
GWAS: genome-wide association study
HPA: hypothalamic–pituitary–adrenal
IL-4: interleukin-4
IL-6: interleukin-6
LHA: lateral hypothalamic area
LS: lateral septum
MSNs: medium spiny neurons
MRs: mineralocorticoid receptors
mRNA: messenger RNA
NAc: nucleus accumbens
NE: norepinephrine
NMDA: *N*-methyl-D-aspartate
PFC: prefrontal cortex
PND: postnatal day
PVN: paraventricular nucleus
SNP: single nucleotide polymorphism
TNF-α: tumor necrosis factor α
UCN I: urocortin I
UCN II: urocortin II
UCN III: urocortin III
VTA: ventral tegmental area
